# Polarization of Human Macrophages by Interleukin-4 Does Not Require ATP-Citrate Lyase

**DOI:** 10.3389/fimmu.2018.02858

**Published:** 2018-12-04

**Authors:** Dmitry Namgaladze, Sven Zukunft, Frank Schnütgen, Nina Kurrle, Ingrid Fleming, Dominik Fuhrmann, Bernhard Brüne

**Affiliations:** ^1^Faculty of Medicine, Institute of Biochemistry I, Goethe-University, Frankfurt, Germany; ^2^Center for Molecular Medicine, Institute for Vascular Signaling, Goethe-University, Frankfurt, Germany; ^3^Department of Medicine 2, LOEWE Center for Cell and Gene Therapy and Frankfurt Cancer Institute, University Hospital Frankfurt, Goethe-University, Frankfurt, Germany; ^4^Project Group Translational Medicine and Pharmacology TMP, Fraunhofer Institute for Molecular Biology and Applied Ecology, IME, Frankfurt, Germany

**Keywords:** macrophage, ATP-citrate lyase, acetyl-CoA, interleukin-4, histone acetylation

## Abstract

Macrophages exposed to the Th2 cytokines interleukin (IL) IL-4 and IL-13 exhibit a distinct transcriptional response, commonly referred to as M2 polarization. Recently, IL-4-induced polarization of murine bone marrow-derived macrophages (BMDMs) has been linked to acetyl-CoA levels through the activity of the cytosolic acetyl-CoA-generating enzyme ATP-citrate lyase (ACLY). Here, we studied how ACLY regulated IL-4-stimulated gene expression in human monocyte-derived macrophages (MDMs). Although multiple ACLY inhibitors attenuated IL-4-induced target gene expression, this effect could not be recapitulated by silencing ACLY expression. Furthermore, ACLY inhibition failed to alter cellular acetyl-CoA levels and histone acetylation. We generated ACLY knockout human THP-1 macrophages using CRISPR/Cas9 technology. While these cells exhibited reduced histone acetylation levels, IL-4-induced gene expression remained intact. Strikingly, ACLY inhibitors still suppressed induction of target genes by IL-4 in ACLY knockout cells, suggesting off-target effects of these drugs. Our findings suggest that ACLY may not be the major regulator of nucleocytoplasmic acetyl-CoA and IL-4-induced polarization in human macrophages. Furthermore, caution should be warranted in interpreting the impact of pharmacological inhibition of ACLY on gene expression.

## Introduction

Macrophages respond to changes in their environment, such as bacterial or viral infection, hormones, cytokines, or nutrients, with remodeling their transcriptome. Consequently, they alter their phenotype, a response known as macrophage polarization (1). Historically, macrophage polarization was described in a dichotomous fashion with a pro-inflammatory response to bacterial lipopolysaccharide in combination with a Th1 cytokine interferon-γ (M1 response) as opposed to an anti-inflammatory response elicited by Th2 cytokines interleukin-4 (IL-4) or IL-13 (M2 response) (2–4).

M1 and M2 responses are remarkably different not only in the signaling pathways involved, but also how they engage variable metabolic pathways (5). While metabolism primarily serves to provide energy and substrates to support macrophage functional responses, e.g., phagocytosis, several metabolites directly affect transcription through epigenetic mechanisms (6). Acetyl-CoA is a metabolite with a distinct role in epigenetic and transcriptional regulation through its widespread use as a substrate for acetylation of histones and other proteins, including transcription factors (7). Although several reactions provide acetyl-CoA for histone acetylation, ATP-citrate lyase (ACLY) is considered to predominantly contribute to nuclear pool of acetyl-CoA (8). ACLY role in epigenetic control was initially described for cancer cells (9), where ACLY critically supports *de novo* lipogenesis and thus, cell proliferation (10, 11). Further studies reported epigenetic regulation through ACLY in adipocytes (9, 12) or myocytes (13).

Recently, ACLY was shown to regulate transcriptional responses to IL-4 in murine bone marrow-derived macrophages (BMDMs) (14). IL-4 triggered the Akt-mediated serine phosphorylation of ACLY, which is supposed to increase ACLY enzymatic activity (15, 16). Accordingly, pharmacological inhibition of Akt or ACLY prevented induction of a subset of IL-4-responsive mRNAs, which was associated with reduced histone acetylation at promoters of ACLY-sensitive genes (14). How ACLY regulates the response of human macrophages to IL-4 is unknown.

Since we previously noticed considerable differences in metabolic requirements of human vs. murine macrophages toward IL-4-induced polarization (17), we questioned the role of ACLY in regulating human monocyte-derived macrophage (MDM) responses to IL-4. Our data suggest that ACLY has little impact on transcriptional regulation of IL-4-responsive genes. Surprisingly, we observed a widespread inhibition of IL-4-induced target gene expression by pharmacological ACLY inhibitors, which persisted in ACLY knockout THP-1 macrophages, suggesting off-target effects of these substances.

## Experimental Procedures

### Cell Culture and Treatment

Human peripheral blood mononuclear cells were isolated from commercially obtained buffy coats (DRK Blutspendedienst Baden-Württemberg-Hessen, Institut für Transfusionsmedizin und Immunhämatologie, Frankfurt, Germany) using Ficoll (Biochrom, cat. no. L6115) density centrifugation. Monocytes were separated from lymphocytes by adherence to plastic after 1 h-incubation in serum-free medium, and differentiated into macrophages by culture in RPMI-1640 medium (Gibco, cat. no. 61870) containing 3% heat-inactivated AB-positive human serum for 7–10 days. THP-1 cells were purchased from ATCC (cat. no. TIB-202) and maintained in RPMI-1640 medium containing 10% fetal bovine serum, penicillin and streptomycin. THP-1 cells were differentiated to macrophages by 24 h-treatment with 50 nM phorbol myristate acetate (Sigma-Aldrich, cat. no. P8139) followed by overnight culture in serum-free medium. Unless indicated otherwise, cells were treated with 5 μM BMS-303141 (cat. no. 4609), 25 μM SB 204990 (cat. no. 4962) (both Tocris), 100 μM MEDICA 16 (cat. no. M5693), 20 mM hydroxycitrate (cat. no. 59847), 5 mM sodium acetate (cat. no. S2889), 5 mM sodium octanoate (cat. no. C5038), 1,2,3-benzene-tricarboxylic acid (cat. no. 51520), CTP inhibitor (cat. no. SML0068) (all Sigma-Aldrich), 5 μg/mL TOFA (cat. no. 10005263) (Cayman), 20 ng/mL IL-4 (cat. no. 200-04), or IL-13 (cat. no. 200-13) (Peprotech).

### RNA Isolation and Analysis

Total RNA from macrophages was isolated using PeqGold RNAPure kit (cat. no. 732-3312, PeqLab) followed by reverse transcription using cDNA Synthesis kit (cat. no. K1542, Fermentas). Quantitative real time PCR (Q-PCR) was carried out on a CFX96 system from Bio-Rad using iQ SYBR green (cat. no. 170-8882, Bio-Rad). Primer sequences are listed in Supplementary Table 1. Target gene expression was calculated using ΔCt method [rel. expression = 2^−(Ct(target)−Ct(reference))^] using expression of β2-microglobulin or GAPDH as reference genes.

### Western Blotting

Total cell lysates were prepared by scraping the cells into Laemmli buffer [2% SDS (cat. no. CN30.3, Carl Roth), 62.5 mM Tris-HCl (cat. no. A1086, Applichem), pH 6.8, 10% glycerol (cat. no. BP229, Fisher), 10 mM DTT (cat. no. 6908.1, Carl Roth)] containing protease inhibitors (Complete, cat. no. 11697498001, Roche) followed by sonification. For histone extraction, cell pellets were lyzed in PBS containing 0.5% Triton X-100 (cat. no. 3051.3, Carl Roth), 5 mM sodium butyrate (cat. no. 303410, Sigma-Aldrich), and protease inhibitors for 10 min on ice. Lysates were centrifuged 0.5 min at 16,000 g, 4°C, and pellets containing nuclei were incubated with 0.2 M HCl (cat. no. 182109, Applichem) overnight. After centrifugation of insoluble material for 10 min at 500 g, 4°C, supernatants were neutralized with NaOH (cat. no. A6579, Applichem), mixed with 5x Laemmli buffer and heated 5 min at 95°C.

Protein lysates from macrophages were run on 7.5–15% polyacrylamide gels and blotted on nitrocellulose membranes. Following primary antibodies were used: pSTAT6 (Y641) (cat. no. #9361), STAT6 (clone D3H4) (cat. no. #5397), pSTAT3 (Y705) (cat. no. #9131), STAT3 (clone 124H6) (cat. no. #9139), pACLY (S454) (cat. no. #4331), histone H3 acK14 (clone D4B9) (cat. no. #7627), histone H3 acK23 (clone D6Y7M) (cat. no. #14932), DDK tag (clone 9A3) (cat. no. #8146) (all Cell Signaling Technology), ACLY (cat. no. 15421-1-AP, Proteintech), histone H3 acK9 (clone Y28) (cat. no. 04-1003, Merck Millipore), histone H3 acK27 (cat. no. C15410174, Diagenode), histone H3 (cat. no. 07-690, Merck Millipore), nucleolin (cat. no. sc-13057, Santa-Cruz). Membranes were incubated with IRDye 700/800-coupled secondary antibodies, scanned and quantified using Odyssey imaging system (Licor).

### siRNA Transfection

Silencing of ACLY was performed using siGENOME SMARTpool (cat. no. M-004915-00-0005, Thermo Fisher Scientific) at 50 nM and Hyperfect transfection reagent (cat. no. 301707, Qiagen). Cells were treated 96 h post-transfection.

### ACLY Overexpression

Human MDMs were transfected with 1 μg DDK-tagged ACLY (cat. no. OHu14076, Genscript) or GFP-encoding plasmids (pmaxGFP, Lonza) using Viromer Red transfection reagent (cat. no. VR-01LB, Lipocalyx) according to manufacturer's instructions. Twenty-four hours after transfection, cells were stimulated with 20 ng/mL IL-4 for 24 h.

### Acetyl-CoA Determination

Cells were rapidly washed with saline, and metabolism was quenched by putting the dishes into liquid nitrogen. Cells were scraped into methanol:water (5:3) mix on ice/dry ice followed by addition of cold chloroform (cat. no. 4432.1, Carl Roth), and vortexing for 10 min at 4°C. Aqueous phase was separated, evaporated, and re-suspended in 10% methanol (cat. no. 32213, Sigma-Aldrich). Acetyl-CoA concentration was determined with a Sciex QTrap5500 mass spectrometer operating in multiple reaction monitoring mode in positive electrospray ionization mode. Chromatographic separation was performed on an Agilent 1290 Infinity LC system (Agilent) using an Acquity HSS T3 column. The mobile phase consisted of (A) water, 10 mM ammonium formate (cat. no. 70221, Sigma-Aldrich), 0.01% ammonia, and (B) methanol, 10 mM ammonium formate, 0.01% ammonia. Elution of analytes was carried out under gradient conditions at a flow rate of 0.3 mL/min going from 2% B to 70% B in 5 min, increasing to 95% B in 1 min, hold 95% B for 0.5 min, and equilibrate at 2% B for 2.5 min. Calibration curve was performed with an authentic standard. All samples and dilutions of the standards were spiked with heavy isotope labeled internal standard containing ^13^C2-acetyl-CoA (cat. no. 658650, Sigma-Aldrich). Acetyl-CoA concentration was determined by reference to the standard. Analyst 1.6.2 and MultiQuant 3.0 (both Sciex), were used for data acquisition and analysis, respectively. Acetyl-CoA amounts in the sample were normalized to sample DNA concentration measured following incubation with Höchst 33342 fluorescent DNA dye (cat. no. B2261, Sigma-Aldrich) on a Tecan fluorescence plate reader.

### CRISPR/Cas 9 Knockout of ACLY in THP-1 Cells

pLentiCRISPRv2 (Addgene: cat. no. #52961) vectors harboring different sgRNAs designed using the benchling software package (Table 1) were obtained by target-specific oligonucleotide annealing using the GoldenGate protocol (18). Cell-free lentiviral supernatants were produced by co-transfection of pLentiCRISPRv2 vectors, gag/pol helper plasmid, and envelope plasmid encoding the glycoprotein of vesicular stomatitis virus into HEK293T cells using the JetPrime transfection reagent (Polyplus Transfection). Seventy-two hours post transfection viral supernatants were harvested and sterile filtered. THP-1 cells were infected with lentiviruses, and transduced cells expressing EGFP were sorted using FACS Aria cell sorter, followed by dilution in cell culture media to obtain single-cell suspensions. Resulting single cell-derived colonies were analyzed for ACLY knockout using Western Blotting.

**Table 1:** Oligonucleotides for cloning of ACLY sgRNA constructs.

hACLY Ex4 s	5^′^-CAC CGc tcg atc aga aag ttc ttg-3^′^
hACLY Ex4 as	5^′^-AAA Cca aga act ttc tga tcg agC-3^′^
hACLY Ex5 s	5^′^-CAC CGc acg tcc aca ccc ccc tcg-3^′^
hACLY Ex5 as	5^′^-AAA Ccg agg ggg gtg tgg acg tgC-3^′^
hACLY Ex6SA s	5^′^-CAC CGa aac tgg cca gaa ttc tag-3^′^
hACLY Ex6SA as	5^′^-AAA Cct aga att ctg gcc agt ttC-3^′^

### Statistical Analysis

Data are presented as means ± S.E. of at least three independent experiments. Data were analyzed by one-way analysis of variance (ANOVA) with Bonferroni *post-hoc* means comparison using GraphPad Prism. Differences were considered statistically significant at *p* < 0.05.

### Ethics

Investigations were conducted in accordance with the ethical standards and according to the Declaration of Helsinki and to the national and international guidelines and have been approved by the authors' institutional review board. The ethics committee of Goethe-University waived the necessity of written informed consent when using the buffy coats from anonymized blood donors.

## Results

**Figure 1:**
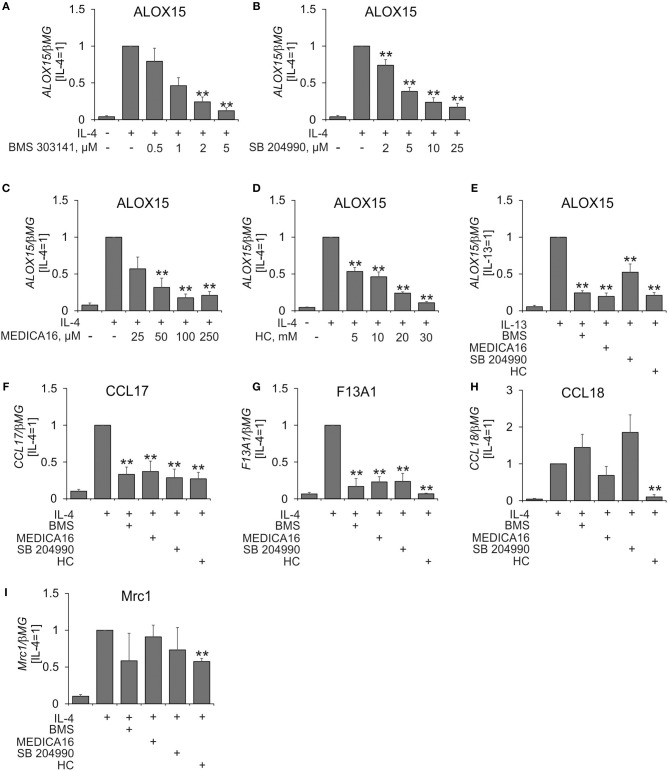
ACLY inhibitors attenuate IL-4-induced target gene expression. **(A–D)** Q-PCR analysis of mRNA expression of ALOX15 in MDMs treated for 1 h with indicated concentrations of BMS 303141 **(A)**, SB 204490 **(B)**, MEDICA 16 **(C)**, or hydroxycitrate (HC) **(D)** prior to 24-h treatment with 20 ng/mL IL-4. **(E–I)** Q-PCR analysis of mRNA expression of indicated genes in MDMs treated for 1 h with 5 μM BMS 303141, 25 μM SB 204490, 100 μM MEDICA 16 or 20 mM HC prior to 24-h treatment with 20 ng/mL IL-13 **(E)** or IL-4 **(F–I)**. **p < 0.01 vs. IL-4 (one-way ANOVA). Data represent mean values ± SE of 4–11 independent experiments.

To investigate the role of ACLY in IL-4-stimulated human macrophage polarization we initially analyzed mRNA expression of arachidonate 15-lipoxygenase (ALOX15) in MDMs in the presence of pharmacological ACLY inhibitors BMS 303141 (19), SB 204990 (20), MEDICA16 (21), and hydroxycitrate (22). Inhibitor concentrations were previously described in the literature. ALOX15 was chosen because of its possible expression sensitivity to metabolic perturbations through regulation by the central metabolic sensor AMP-activated protein kinase (23). MDMs were pre-incubated with ACLY inhibitors for 1 h followed by 24 h-treatment with IL-4 in the presence of inhibitors. As shown in Figures 1A–D, all ACLY inhibitors concentration-dependently suppressed IL-4-induced ALOX15 mRNA expression. Similarly, ACLY inhibitors prevented ALOX15 mRNA induction in MDMs treated with the Th2 cytokine IL-13 (Figure 1E).

A previous study showed that ACLY was necessary for IL-4-induced expression of a subset of IL-4 target genes in murine macrophages (14). To assess the ubiquitous nature of ACLY in affecting IL-4-stimulated gene expression in MDMs we analyzed mRNA expression of several well described IL-4 targets, e.g., CCL17, F13A1, CCL18, and MRC1 (CD206) (24). Whereas, IL-4-stimulated CCL17 and F13A1 mRNA expression was uniformly inhibited by ACLY inhibitors, this was not the case for CCL18 and MRC1 (Figures 1F–I). Apparently, the sensitivity toward ACLY inhibition is IL-4 target gene-specific. Remarkably, analyzing the list of top 50 IL-4 responsive genes sensitive to ACLY inhibition in murine BMDMs (14), we found that only 5 genes (CCL17, CAMK2A, PHF19, ALDH1A2, ITGAX) were induced by IL-4 more than 1.5-fold in MDMs (25). This highlights the differences between the transcriptional responses toward IL-4 in human and murine systems. Except for CCL17, none of these genes showed more than two-fold induction by IL-4 or ACLY inhibitor sensitivity in quantitative PCR analyses (data not shown).

**Figure 2:**
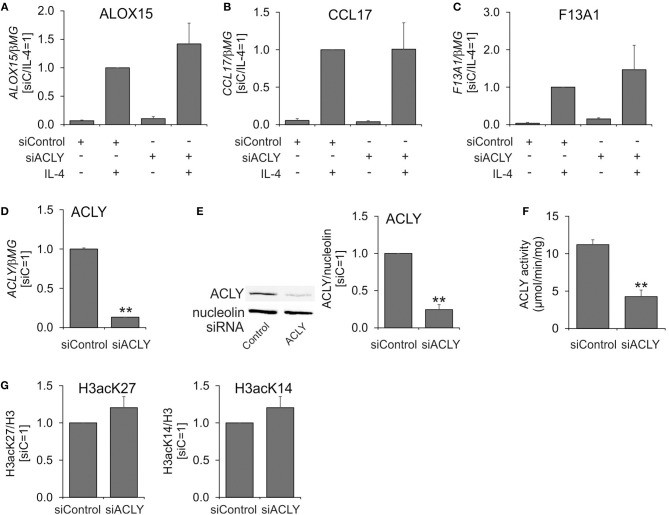
An ACLY knockdown does not recapitulate the phenotype of ACLY inhibitor-treated MDMs. **(A–D)** Q-PCR analysis of mRNA expression of ALOX15, CCL17, F13A1, and ACLY in MDMs transfected with 50 nM ACLY siRNA for 96 h prior to 24-h treatment with 20 ng/mL IL-4. **(E–G)** Western blot analysis of ACLY protein expression **(E)**, assay of enzymatic activity **(F)**, and Western blot analysis of histone H3 acetylation at K27 and K14 **(G)** 96 h post-transfection with ACLY siRNA. **p < 0.01 vs. siControl (one-way ANOVA). Data represent mean values ± SE of 3–4 independent experiments.

Next, we assessed the effect of silencing ACLY mRNA expression on IL-4-induced MDM polarization. For this, we treated MDMs for 96 h with control or ACLY siRNAs prior to 24 h-treatment with IL-4. Surprisingly, a knockdown of ACLY failed to reproduce the effect of ACLY inhibitors on IL-4-stimulated gene expression (Figures 2A–C), despite of a 90% decrease of ACLY mRNA (Figure 2D), an 80% reduced ACLY protein expression (Figure 2E), and a 60% decrease in ACLY enzymatic activity (Figure 2F). We also did not observe any significant changes of IL-4-induced target gene mRNA expression upon overexpression of ACLY in MDMs (Supplementary Figure 1). Since the impact of ACLY on gene expression is linked to reduced acetylation of histone proteins in macrophages (14) and other cells (9, 13), we analyzed acetylation of lysine 27 and 14 on histone H3. Acetylation of H3K14 and H3K27 was previously shown to respond to alterations of ACLY activity (12, 26). A knockdown of ACLY failed to alter acetylation of H3K14 and H3K27 (Figure 2G). These data suggest that ACLY silencing in primary human MDMs does not recapitulate the effect of pharmacologic ACLY inhibition on IL-4-induced gene expression.

**Figure 3:**
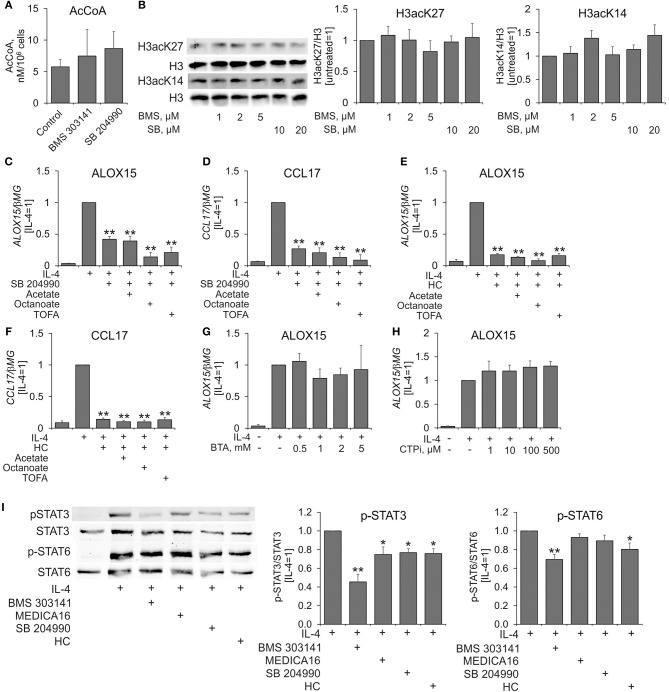
Effects of ACLY inhibitors on acetyl-CoA levels, histone acetylation, and IL-4-induced STAT6 and STAT3 activation in human MDMs. **(A)** LC-MS analysis of acetyl-CoA levels in MDMs treated with 5 μM BMS 303141 and 10 μM SB 204990 for 24 h. **(B)** Western blot analysis of histone H3 acetylation at K14 and K27 in MDMs treated with indicated concentrations of BMS 303141 and SB 204990 for 24 h. **(C–F)** Q-PCR analysis of mRNA expression of ALOX15 and CCL17 in MDMs pre-treated with acetate, octanoate and TOFA for 1 h prior to treatments with SB 204490, hydroxycitrate, and IL-4 for 24 h. **(G,H)** Q-PCR analysis of mRNA expression of ALOX15 in MDMs pre-treated with indicated concentrations of BTA **(G)** or CTPi **(H)** for 1 h prior to treatment with IL-4 for 24 h. **(I)** Western blot analysis of STAT3 and STAT6 tyrosine phosphorylation in MDMs pre-treated with ACLY inhibitors 1 h prior to 0.5 h-treatment with IL-4. *p < 0.05, **p < 0.01 vs. IL-4 (one-way ANOVA). Data represent mean values ± SE of 3–4 independent experiments.

Considering these discrepancies, we decided to investigate the impact of ACLY inhibitors on human macrophage metabolism in more detail. Surprisingly, we did not find any differences in the levels of acetyl-CoA in cells treated with BMS 303141 or SB 204990 for 24 h (Figure 3A). Accordingly, treating MDMs with ACLY inhibitors for 24 h did not affect histone H3 acetylation at Lys14 or Lys27 (Figure 3B). Next, we exploited treatments known to increase nucleocytosolic levels of acetyl-CoA and histone acetylation. In these experiments, MDMs were pre-incubated with acetate (9), inhibitor of acetyl-CoA carboxylase TOFA (27), or octanoate (28), for 1 h prior to treatments with SB 204990, hydroxycitrate, and IL-4. As Figures 3C–F show, acetate, TOFA, and octanoate failed to reverse inhibition of IL-4-stimulated ALOX15 and CCL17 expression by SB 204990 or hydroxycitrate. Alternatively, we aimed to block the transport of the ACLY substrate citrate from mitochondria to the cytosol by pre-treating MDMs with different concentrations of pharmacological inhibitors of the mitochondrial citrate carrier SLC25A1 1,2,3-benzene-tricarboxylic acid (BTA) (29) and 4-chloro-3-{[(3-nitrophenyl)amino]sulfonyl}benzoic acid (CTPi) (30) for 1 h prior to 24 h-treatment with IL-4. Neither BTA, nor CTPi influenced IL-4-stimulated gene expression (Figures 3G,H). These results indicate that the impact of ACLY inhibitors on IL-4-induced gene expression may be unrelated to regulation of nucleocytosolic acetyl-CoA.

We then investigated the influence of ACLY inhibitors on initial steps of IL-4-induced signal transduction by pre-incubating MDMs with inhibitors for 1 h followed by 0.5 h-treatment with IL-4. ACLY inhibitors did not affect IL-4-triggered tyrosine phosphorylation of STAT6 with the exception of BMS 303141 and hydroxycitrate (Figure 3I). We noticed 25–30% inhibition of STAT3 tyrosine phosphorylation in ACLY inhibitor-exposed, IL-4-stimulated MDMs (Figure 3I). Whereas, STAT3 phosphorylation at Ser727 and acetylation at Lys685 were reported to affect STAT3 transcriptional activity, neither IL-4, nor ACLY inhibitors influenced these post-translational modifications in our system (data not shown).

**Figure 4:**
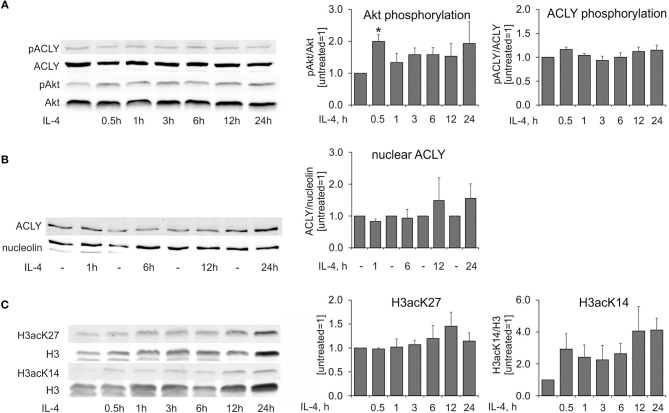
IL-4 does not induce ACLY phosphorylation in human MDMs. **(A–C)** Western blot analysis of ACLY and Akt phosphorylation **(A)**, nuclear ACLY levels **(B)**, and histone H3 acetylation at K27 and K14 **(C)** in MDMs treated with 20 ng/mL IL-4 for indicated times. Data represent mean values ± SE of 3–4 independent experiments. *p < 0.05 vs. untreated (one-way ANOVA).

Murine BMDMs responded to IL-4-stimulation with an Akt-dependent increase of ACLY phosphorylation at Ser454, which exhibited a delayed kinetics as compared with IL-4-induced Akt phosphorylation (14). We followed kinetics of Akt and ACLY phosphorylation in MDMs stimulated with IL-4 for different times. In our hands, Akt phosphorylation at Ser473 was transiently increased in IL-4-stimulated human MDMs (Figure 4A). However, we noticed very modest differences in ACLY phosphorylation during the time course of IL-4 treatment (Figure 4A). ACLY is known to be partially present in the nuclear fraction of different cancer cell lines (9). We also observed ACLY in the nuclear fraction of human MDMs, although most of the enzyme was located in the cytosol. IL-4 did not cause any alteration of ACLY localization (Figure 4B). We failed to detect ACLY phosphorylation in the nuclear fraction in response to IL-4. Finally, we tracked histone acetylation in MDMs stimulated with IL-4 for different times. Whereas, lysine 27 of histone H3 exhibited no significant changes in acetylation, K14 acetylation increased within 0.5 h of IL-4 treatment and remained elevated up to 24 h (Figure 4C). Collectively, these observations indicate that in human MDMs ACLY does not seem to be regulated by phosphorylation or nuclear transport. This is in contrast to results obtained in the murine system.

**Figure 5:**
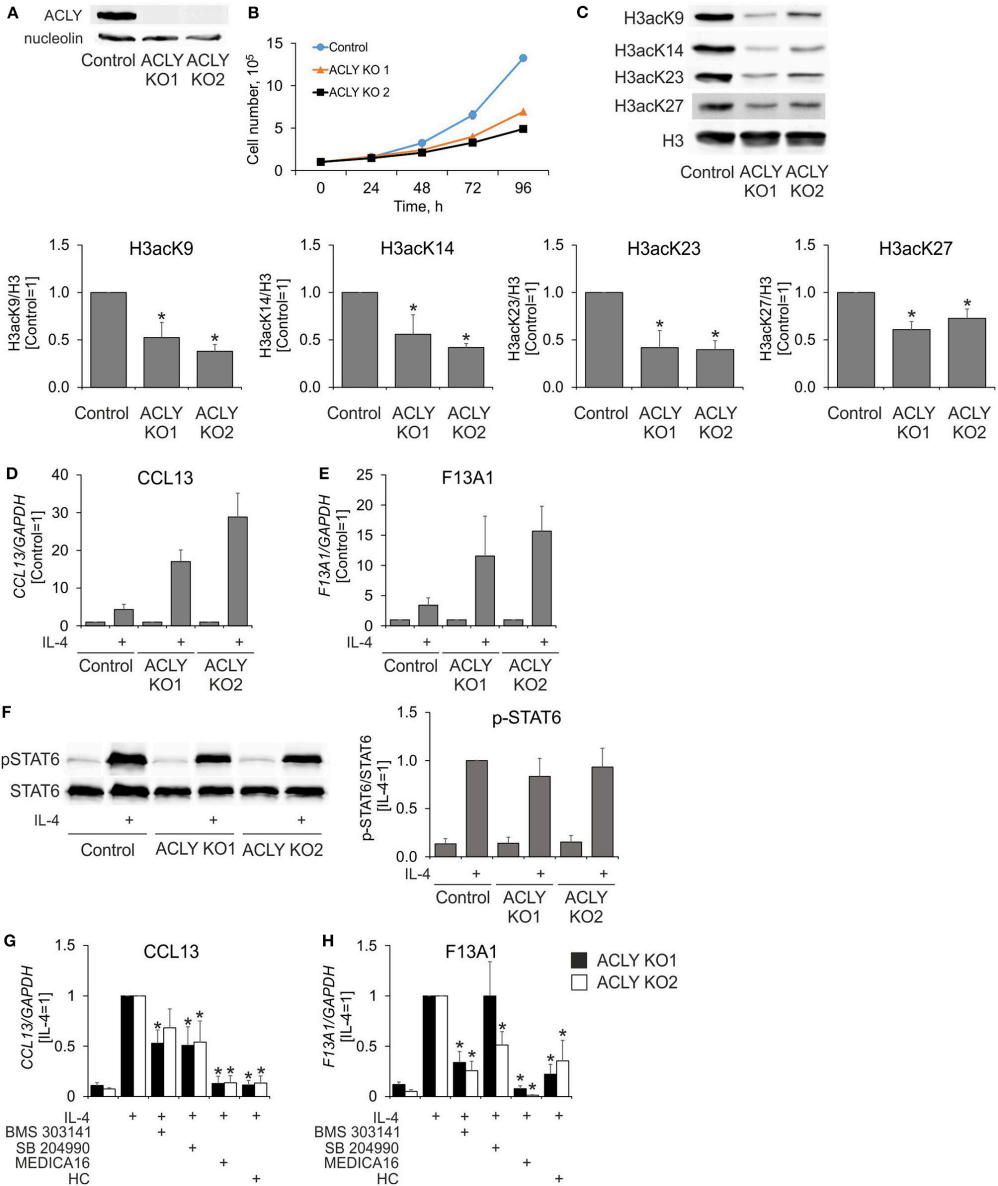
ACLY inhibitors suppress IL-4-induced gene expression in ACLY knockout THP-1 cells. **(A,B)** Western blot analysis of ACLY protein expression **(A)** and growth curves **(B)** of control and ACLY knockout (ACLY KO) THP-1 cells. **(C)** Western blot analysis of histone H3 acetylation at K9, K23, K27, and K14 in control or ACLY KO THP-1 macrophages. **(D,E)** Q-PCR analysis of IL-4—induced mRNA expression of CCL13 and F13A1 in control or ACLY KO THP-1 macrophages. **(F)** Western blot analysis of STAT6 phosphorylation in control or ACLY KO THP-1 macrophages. **(G,H)** Q-PCR analysis of mRNA expression of CCL13 and F13A1 in ACLY KO THP-1 macrophages stimulated with IL-4 in the presence of BMS 303141, SB 204990, MEDICA16 or HC. *p < 0.05, vs. Control or IL-4 (one-way ANOVA). Data represent mean values ± SE of 4–6 independent experiments.

An ACLY knockdown in human MDMs left substantial amounts of residual ACLY activity. Therefore, we proceeded to create a knockout of ACLY in human myeloid THP-1 cells, which upon differentiation resemble human MDMs. Although they have a defect in IL-4 receptor signaling through the absence of a common gamma receptor chain (31), THP-1 cells retain transcriptional responses to IL-4 stimulation and are used to investigate IL-4-induced human macrophage polarization (32). Figure 5A shows the complete absence of ACLY protein in ACLY knockout THP-1 cells. As expected, ACLY-deficient THP-1 cells exhibited delayed growth, likely through deficiencies in *de novo* lipogenesis (Figure 5B). In contrast to primary macrophages, ACLY knockout THP-1 macrophages showed reduced levels of histone H3 acetylation on lysines 9, 14, 23, and 27 (Figure 5C). However, ablation of ACLY did not prevent the ability of THP-1 to respond to 24 h IL-4-stimulation with increased gene expression (Figures 5D,E). This was also reflected by intact phosphorylation of STAT6 after 0.5 h IL-4-treatment in ACLY knockout cells (Figure 5F). Most strikingly, pre-incubating ACLY knockout THP-1 cells with ACLY inhibitors for 1 h still suppressed IL-4-induced mRNA expression of CCL13 and F13A1 (Figures 5G,H). These data strongly indicate that ACLY inhibitors suppress IL-4-induced gene expression independently of ACLY through off-target effects.

## Discussion

ACLY is thought to link metabolism and epigenetic control of transcription through its provision of acetyl-CoA for nuclear histone acetylation (8). Strikingly, our study suggests that ACLY has little, if any, influence on IL-4-induced transcriptional responses and histone acetylation in human MDMs. Our data thus contrast with observations in murine BMDMs, where ACLY was shown to significantly contribute to the induction of at least a subset of the IL-4-sensitive transcriptome by increasing histone acetylation (14). Moreover, we show that several commonly used pharmacological ACLY inhibitors influence IL-4-induced gene expression even in the absence of ACLY, strongly pointing to off-target effects of these drugs. This warrants caution in interpretation of the previous study (14) as well as other reports relying on use of ACLY inhibitors.

Which differences between our and murine BMDM system may explain the observed discrepancies? Obviously, species difference can be a major factor. Studies from our and other groups show that murine and human macrophages differ not only in how their transcriptome is altered in response to IL-4 (25, 33), but also how their metabolism controls IL-4-dependent gene expression (17). We now observe that initial steps in signal transduction downstream of the IL-4 receptor differ between human and murine systems. Thus, human MDMs do not show Akt-dependent ACLY phosphorylation in response to IL-4. Akt activation in our system is only transient in contrast to prolonged activation in BMDMs (14). Whether this relates to differences in insulin receptor substrate 2 engagement by the IL-4 receptor, which is thought to be responsible for Akt activation by IL-4 (31), remains to be investigated. Another difference is sustained proliferation in response to macrophage colony-stimulating factor and IL-4 (34), which was noticed in BMDMs (14). Since high ACLY activity is a pre-requisite for ongoing proliferation of cells through its contribution to *de novo* lipogenesis (10), proliferating macrophages may be characterized by increased ACLY activity, exerting greater influence on nuclear acetyl-CoA and, consequently, histone acetylation. In contrast, our experimental setup employed fully differentiated macrophages, which do not proliferate. Negligible *de novo* lipogenesis was evidenced by the failure to incorporate C13-carbon from C13-labeled glucose to cellular palmitate (data not shown). Thus, the role of ACLY in metabolism and epigenetic regulation of terminally differentiated human macrophages remains unclear and warrants further research. Of note, ACLY may have greater impact during human macrophage differentiation, since this process is characterized by a temporary rise of *de novo* lipogenesis (35). Another proposed function of ACLY in macrophages, based on observations in U937 cell line, is the provision of substrates for increased synthesis of bioactive mediators, such as prostaglandin E_2_, nitric oxide, or reactive oxygen species, in response to pro-inflammatory stimuli (36), which remains to be validated in primary cells.

Based on our studies, a critical unresolved question is, which enzymatic system provides nuclear acetyl-CoA for histone acetylation in human MDMs. Whereas, our data show that ACLY definitely contributes to histone acetylation in the acute myeloid leukemia cell line THP-1, we obtained no evidence for such a behavior in human MDMs. Several alternative sources of nuclear acetyl-CoA are described. The major source is conversion of acetate to acetyl-CoA by the action of nucleocytosolic acyl-CoA synthetase short-chain family member 2 (ACSS2). ACSS2 contributes to histone acetylation in cancer cells, especially under hypoxia or glucose deprivation (37, 38), and can also participate in recycling of acetate released from histones by the action of histone deacetylases (39). Acetyl-CoA can also be synthesized in the nucleus through nuclear translocation of the pyruvate dehydrogenase complex (40, 41). An alternative cytosolic acetyl-CoA generation system, involving coordinated actions of mitochondrial succinyl-CoA:3-ketoacid-CoA transferase and cytosolic acetoacetyl-CoA synthetase and acetyl-CoA acyltransferase activities was described for pancreatic beta-cells (42). Finally, activity of carnitine acetyl-CoA transferase and other uncharacterized pathways were proposed to link mitochondrial and nucleocytosolic acetyl-CoA (28, 43, 44). Which of these pathways contributes to nucleocytosolic acetyl-CoA in human MDMs remains the topic of current investigation.

Our results also highlight the notorious proneness of pharmacological inhibitors to off-target effects, which is in our case particularly remarkable, since we used structurally dissimilar substances (hydroxylated citrate, tertramethylated long chain dicarboxylic fatty acid, tricyclic aromatic sulfonamide, and a dychlorphenylhexyl-substituted hydroxylated derivative of tetrahydrofuranacetic acid). Off-target effects of ACLY inhibitors on IL-4-stimulated gene transcription could only be revealed using ACLY knockout cell line. Our findings also point to the limitation of studying human primary cells, since ACLY knockdown leaves substantial residual activity left whereas pharmacological inhibitors are unsuitable due to off-target activities. Whether induced pluripotent stem cell—derived macrophages, where creation of knockout models is possible (45), will be a better suitable model for studying metabolic regulation of macrophage polarization as compared to acute myeloid leukemia THP-1 cells, should be revealed by future investigation.

## Author Contributions

DN designed the study, performed the experiments, and wrote the manuscript. SZ and IF contributed to acetyl-CoA measurements. FS, NK, and DF contributed to CRISPR/Cas9 THP-1 knockout cell line creation. BB contributed to study design and edited the final manuscript. All authors contributed to manuscript revision, read, and approved the submitted version.

### Conflict of Interest Statement

The authors declare that the research was conducted in the absence of any commercial or financial relationships that could be construed as a potential conflict of interest.

## Abbreviations

ACLY: ATP-citrate lyase
ALOX15: arachidonate 15-Lipoxygenase
BMDM: bone marrow-derived macrophage
IL: interleukin
MDM: monocyte-derived macrophage.
