# Supplementary material for: Polarization of Human Macrophages by Interleukin-4 Does Not Require ATP-Citrate Lyase
Source: Front Immunol. 2018 Dec 4;9:2858. doi: 10.3389/fimmu.2018.02858 (PMC6290342; doi:10.3389/fimmu.2018.02858)
Supplement: Supplementary file 1 [file Data_Sheet_1.PDF]

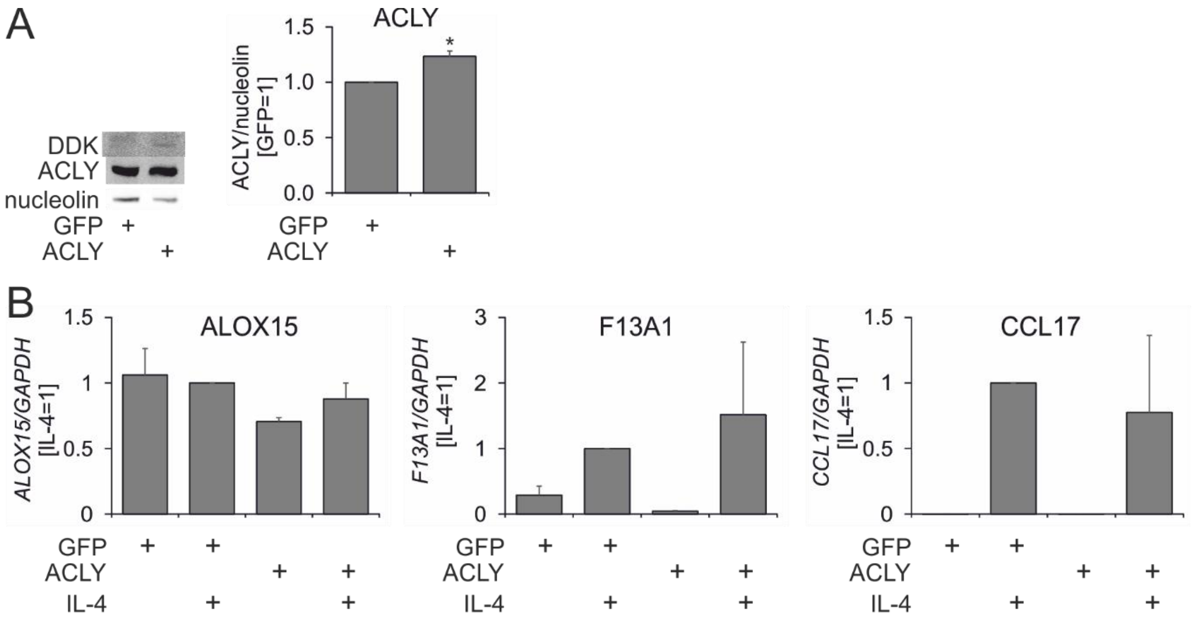

Supplementary Figure 1. ACLY overexpression does not affect IL-4-induced gene expression in MDMs. (A) ACLY protein expression in GFP and ACLY-overexpressing MDMs. **(B)** IL-4 - induced mRNA expression of ALOX15, F13A1 and CCL17 in GFP and ACLY-overexpressing MDMs. *, $p<0.05$, vs. GFP. Data represent mean values ± SE of 3 independent experiments.

Table S1: Primer sequences

ALOX15 forward	5‘-TGGAAGGACGGGTTAATTCTGA-3‘
ALOX15 reverse	5‘-GCGAAACCTCAAAGTCAACTCT -3‘
CCL17 forward	5‘-TTCTCTGCAGCACATCCACG -3‘
CCL17 reverse	5‘-TGTTGGGGTCCGAACAGATG -3‘
CCL18 forward	5‘-CCCAGCTCACTCTGACCACT -3‘
CCL18 reverse	5‘-GTGGAATCTGCCAGGAGGTA -3‘
CCL13 forward	5‘-GGAGATCTGTGCTGACCCAA -3‘
CCL13 reverse	5‘-AGCATAGAAGAGGAGGCCAGA -3‘
F13A1 forward	5‘-TCAGAAACTTCCAGGACCGC -3‘
F13A1 reverse	5‘-AGGTGAACGCTCGTGACATT -3‘
MRC1 forward	5‘-TTCCTTTGGACGGATGGACG -3‘
MRC1 reverse	5‘-CCTCGTTTACTGTCGCAGGT -3‘
GAPDH forward	5‘-ACAACTTTGGTATCGTGGAAGG -3‘
GAPDH reverse	5‘-GCCATCACGCCACAGTTTC -3‘
βMG forward	5‘-GAGGCTATCCAGCGTACTCCA -3‘
βMG reverse	5‘-CGGCAGGCATACTCATCTTTT -3‘
